# Women Doctors

**Published:** 1920-07-31

**Authors:** 


### THE PUBLIC WELL-BEING.

### Women Doctors.

### TWO POINTS OF VIEW.

THOUGH women doctors have evidently come to stay, and the many prejudices against their admission to the ranks of the medical profession are fast disappearing, the following communications which we have received, both from women, show that among women themselves two strongly-marked points of view still exist. One of our correspondents, a Scottish lady, writes:

"I have noticed lately among several of my male medical acquaintances a tendency to speak in subtle disparagement of their colleagues of the other sex in the same profession. It is not so much what they say as the tone and manner in which they say it—the slight shrug of the shoulders, the contemptuous inflection of the voice, the hint of sarcasm. Why is this? Is it because they are afraid of the increasing skill and knowledge of women doctors, their growing favour with patients of their own sex, and particularly the threat of loss of practice in maternity cases? On these occasions I have never lost the opportunity of upholding vigorously the cause of the women doctors, because my own experience justifies it.

"Up to the time of my first pregnancy I had been attended in illness by men doctors, and let me confess at once that I was never free from some timidity in approaching them and from some diffidence in speaking to them with candour and frankness on the intimate details of one's personal symptoms. They on their part were never conspicuous for their tact or sympathy in helping me to talk naturally, and it is not surprising, therefore, that I was not as a rule particularly impressed by the degree of their apprehension of the nature and needs of my illness. For the birth of my child I was advised to go to a woman doctor, who was married and herself a mother, and who had a great reputation for her skill and sympathy. It was a happy inspiration. I shall remember with gratitude all my life the sense and relief with which I was able to tell her everything, her unfailing understanding, her wise and intimate counsel, and the cool confident skill with which she attended me at confinement and afterwards. I am sure she conferred a real benefit on the health of my child as well as on my own. She has made a deep study of obstetrics and is a most valuable surgeon. She has won her way from a very humble start in face of the dogged opposition of the opposite sex. My advice to women, based on my own experience, is 'Go to a woman doctor every time.'"

#### OLD-FASHIONED ENOUGH.

The other letter came from a Somersetshire lady, who, *apropos* of another matter not relevant to the question here discussed, declares that in a serious illness with which she was stricken down while staying in another part of England she had to get hurried medical assistance, and was attended by a woman doctor, "who lost her head and nearly lost me my life." "I am old-fashioned enough to be convinced," she adds, "that there is nothing to equal the old-fashioned *man* doctor. Women have neither the temperament nor the nerve—nor have they normally the physical strength—that form an essential part of a general practitioner's equipment. I have not the same confidence, anyhow, in the woman doctor as in a man, and should be nervous to the point of distraction if in any future sickness I were compelled to accept the ministrations of a member of my own sex, however good her qualifications might be. That, I know, is the view held equally by large numbers of women."

If we may be permitted to intervene in this delicate issue it is only to say that the "temperament" and the "nervousness" are more likely to be found on the side of the woman patient than in the woman doctor. In all probability the purely sex distinctions revealed above will in course of time be reduced to the proper level of importance. They represent a human equation which cannot altogether be dismissed from consideration. On the other hand, the doctors of the future will be less regarded by women patients from the standpoint of sex, but more and more on the grounds of their skill, judgment, and personality, irrespective of whether God has made them male or female.

